# Use of PET/CT to aid clinical decision-making in cases of solitary pulmonary nodule: a probabilistic approach

**DOI:** 10.1590/0100-3984.2019.0034

**Published:** 2020

**Authors:** Felipe Alves Mourato, Ana Emília Teixeira Brito, Monique Sampaio Cruz Romão, Renata Guerra Galvão Santos, Cristiana Altino de Almeida, Paulo José de Almeida Filho, Aline Lopes Garcia Leal

**Affiliations:** 1 Real Hospital Português de Beneficência em Pernambuco, Recife, PE, Brazil.

**Keywords:** Solitary pulmonary nodule, Positron emission tomography, Decision support techniques, Clinical decision-making, Medical oncology, Pulmonary medicine

## Abstract

**Objective:**

To determine the frequency with which ^18^F-FDG-PET/CT findings change the probability of malignancy classification of solitary pulmonary nodules.

**Materials and Methods:**

This was a retrospective analysis of all ^18^F-FDG-PET/CT examinations performed for the investigation of a solitary pulmonary nodule between May 2016 and May 2017. We reviewed medical records and PET/CT images to collect the data necessary to calculate the pre-test probability of malignancy using the Swensen model and the Herder model. The probability of malignancy was classified as low if < 5%, intermediate if 5-65%, and high if > 65%. Cases classified as intermediate in the Swensen model were reclassified by the Herder model.

**Results:**

We reviewed the records for 33 patients, of whom 17 (51.5%) were male. The mean age was 68.63 ± 12.20 years. According to the Swensen model, the probability of malignancy was intermediate in 23 cases (69.7%). Among those, the application of the Herder model resulted in the probability of malignancy being reclassified as low in 6 (26.1%) and as high in 8 (34.8%).

**Conclusion:**

^18^F-FDG-PET/CT was able to modify the probability of malignancy classification of a solitary pulmonary nodule in more than 50% of the cases evaluated.

## INTRODUCTION

A solitary pulmonary nodule (SPN) is a round or oval pulmonary opacity of up to 3 cm in diameter, surrounded by normal lung parenchyma, that is not accompanied by pleural effusion, pneumonia, or adenopathy^(1)^. A SPN is found in 0.09-2% of all chest X-rays^(2)^ and in up to 51% of chest tomography scans in populations at high risk for lung cancer^(3)^. The etiological investigation of a SPN depends on its pre-test probability of malignancy^(1,4,5)^. Cases with low probability of malignancy (< 5%) are usually managed by active surveillance, whereas those with high probability (> 65%) biopsy or surgery is indicated^(1,5)^. Cases with intermediate probability of malignancy are usually biopsied for diagnostic clarification^(6)^. However, biopsy is an invasive procedure that can lead to a significant number of false-negative results^(7)^. The use of PET/CT with ^18^F-FDG (^18^F-FDG-PET/CT) can help to define the management of intermediate cases by reclassifying them as having low, intermediate, or high probability of malignancy^(1)^, thus reducing the number of invasive procedures and the total cost of treatment^(8)^. However, there are no studies evaluating the frequency with which ^18^F-FDG-PET/CT determines a change in the probability of malignancy classification of SPNs, from intermediate to low or high.

Because ^18^F-FDG is a glucose analogue and tumor cells usually have an increased glycolytic metabolism-due to an increased glycolytic enzyme activity and to the overexpression of glucose transporters^(9)^-the tumor cells have enhanced ^18^F-FDG uptake, which makes them visible on ^18^F-FDG-PET/CT images. Therefore, it is possible to distinguish benign from malignant lesions in various conditions, including SPNs. Although the uptake in malignant lesions is usually more intense, the distinction is not always clear, because some benign conditions can also present increased ^18^F-FDG uptake, leading to false-positive results, whereas some malignant lesions may not have affinity for ^18^F-FDG^(10)^.

The SPN pre-test probability of malignancy is defined on the basis of clinical and radiological data. The first model, proposed by Swensen et al.^(11)^, is known as the Mayo Clinic model. That model includes the following clinical variables: age; previous or current smoking; history of extrathoracic cancer; nodule size; presence of nodule spiculation; and nodule location. In 2005, Herder et al.^(12)^ described a new method for calculating the pre-test probability of malignancy. Their model includes all variables of the Mayo Clinic model plus an assessment of ^18^F-FDG uptake by the SPN, as seen on ^18^F-FDG-PET/CT. This new approach resulted in higher accuracy levels^(13)^. However, ^18^F-FDG-PET/CT is performed in cases classified by the Mayo Clinic model as intermediate probability only^(1)^. Therefore, ^18^F-FDG-PET/CT is expected to reclassify intermediate probability cases as low (< 5%) or high (> 65%) probability, probabilistically defining the best management for each case.

The purpose of this study was to determine how often ^18^F-FDG-PET/CT changes the SPN pre-test malignancy classification, based on the probability models described, during the clinical decision-making process.

## MATERIALS AND METHODS

This was a retrospective study based on the analysis of all ^18^F-FDG-PET/CT scans performed between May 1, 2016 and May 31, 2017 at a nuclear medicine center. All ^18^F-FDG-PET/CT scans that were requested for SPN investigation were included. The retrospective analysis of the data was approved by the local research ethics committee (Reference no. 76305317.4.0000.5199).

All ^18^F-FDG-PET/CT scans were performed with the same device (Biograph 16; Siemens Healthcare, PA, USA) approximately 60 min after intravenous administration of 3.7-4.8 MBq/kg of ^18^F-FDG. The PET images were obtained from the base of the skull to the proximal third of the lower limbs in three-dimensional mode, each body segment position being scanned for 3 min. The images obtained were processed by iterative reconstruction (two iterations of eight subsets with a Gaussian filter). Computed tomography (CT) image acquisition parameters included a slice thickness of 5 mm, a voltage of 120 kV, and no intravenous contrast administration. In addition, a high resolution chest CT scan was performed during a maximal inspiratory breath hold in all patients.

Patient medical records were reviewed, and the following data were collected: gender; age; previous or current smoking; history of extrathoracic cancer; results of the SPN biopsy; and results of follow-up chest CT (cases in which a nodule had been stable for two years or had disappeared were considered negative). The images acquired in the dedicated chest CT performed as part of the ^18^F-FDG-PET/CT were then analyzed to determine the diameter of the nodule and whether or not there was spiculation. The PET and CT images were fused and evaluated. The nodule uptake of ^18^F-FDG was classified, by its maximum standardized uptake value (SUVmax), as follows^(13)^: discrete, when the SUVmax was ≤ 2.5; moderate, when the SUVmax was 2.6-10; or intense, when the SUVmax was > 10.

### Calculation of the pre-test probability of malignancy according to the Mayo Clinic model

The Mayo Clinic model determines the SPN probability of malignancy with the following formula^(11)^: $\mathrm{PM}=1/\left(1+e^{-x}\right)$, where *PM* is the probability of malignancy and *x* = −6.8272 + 0.0391 × (age in years) + 0.7917 × (previous or current smoking) + 1.3388 × (history of extrathoracic cancer) + 0.1274 × (SPN diameter in mm) + 1.0407 × (presence of spiculation) + 0.7838 × (SPN located in the upper lobe). Previous or current smoking, history of extrathoracic cancer, presence of spiculation, and SPN location in the upper lobe are taken as dichotomous variables, meaning that when they are absent they get a score of zero and when they are present they get a score of one.

### Calculation of the pre-test probability of malignancy according to the Herder model

The Herder model determines the probability of malignancy by making use of the Mayo Clinic model together with the ^18^F-FDG-PET/CT results, according to the following formula^(12)^: $\mathrm{PM}=1/\left(1+e^{-x}\right)$, where *PM* is the probability of malignancy and *x* = −4.739 + 3.691 × (Mayo Clinic probability) + 2.322 × (discrete nodule uptake) + 4.617 × (moderate nodule uptake) + 4.771 × (intense nodule uptake). The presence of discrete, moderate, and intense uptake are treated as dichotomous variables, meaning that if it is absent it gets a score of zero and if it is present it gets a score of one.

### Ability of ^18^F-FDG-PET/CT to identify high or low probability in cases originally classified as having intermediate probability

Nodules classified by the Mayo Clinic model as having an intermediate probability of malignancy were included in this analysis. In those cases, the probability of malignancy was reclassified according to the Herder model as low (< 5%), intermediate (5-65%), or high (> 65%). ^18^F-FDG-PET/CT was considered a determinant of the best course of action in the cases reclassified as having a low or high probability, although not in the cases that were not reclassified.

### Statistical analysis

Continuous variables are expressed as mean and standard deviation, whereas categorical variables are expressed as absolute and relative frequencies. To assess the ability of ^18^F-FDG-PET/CT to inform practice, the cases were divided into inconclusive (intermediate probability of malignancy) or conclusive (low or high probability of malignancy). We used McNemar’s test to compare the proportions in each model. An additional test was performed only in the cases classified as intermediate probability by the Mayo Clinic model. We used confidence interval (CI) to describe the results of this analysis and considered a *p* < 0.05 as significant. MedCalc Software, version 18.2.1 (MedCalc Software bvba, Ostend, Belgium) was used in the analyses.

## RESULTS

A total of 33 patients was enrolled in this study. The mean age was 68.63 ± 12.20 years, and 17 (51.5%) of the patients were male. The mean probability of malignancy was 43.2 ± 25.6% with the Mayo Clinic model and 47.1% ± 38.9% with the Herder model. The other variables considered in this study are detailed in Table 1.

**Table 1:** Characteristics of the patients and SPNs in our study sample.

Variable	Value
Gender, n (%)	
Male	17 (51.5%)
Female	16 (48.5%)
Age (years), mean ± SD	68.63 (± 12.20)
Current or former smoker, n (%)	26 (78.8%)
History of extrathoracic cancer, n (%)	3 (9.1%)
SPN diameter (cm), mean ± SD	1.52 (± 0.61)
SPN spiculation, n (%)	18 (54.5%)
SPN in the upper lobe, n (%)	20 (60.6%)
SPN 18F-FDG uptake (SUVmax), n (%)	
None	10 (30.3%)
< 2.5	8 (24.2%)
2.5-10.0	13 (39.4%)
> 10.0	2 (6.1%)

**Figure 1:**
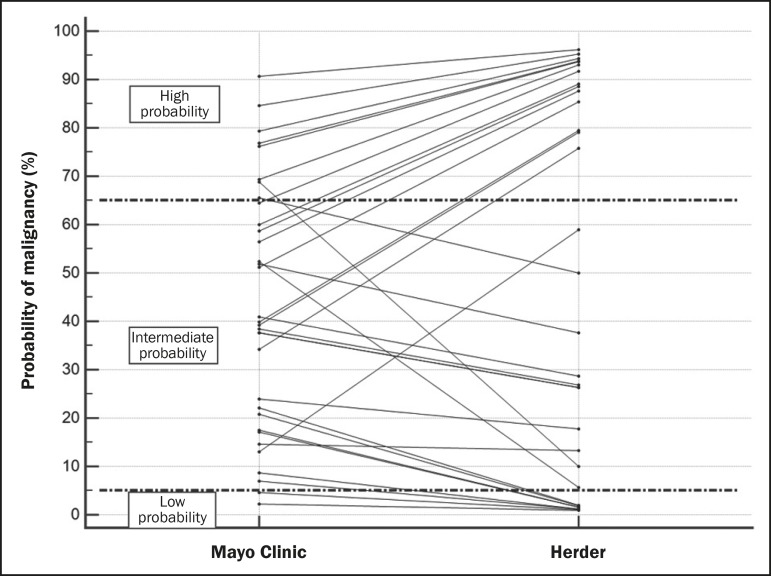
Variation in the probability of malignancy classification per patient according to each model.

**Figure 2:**
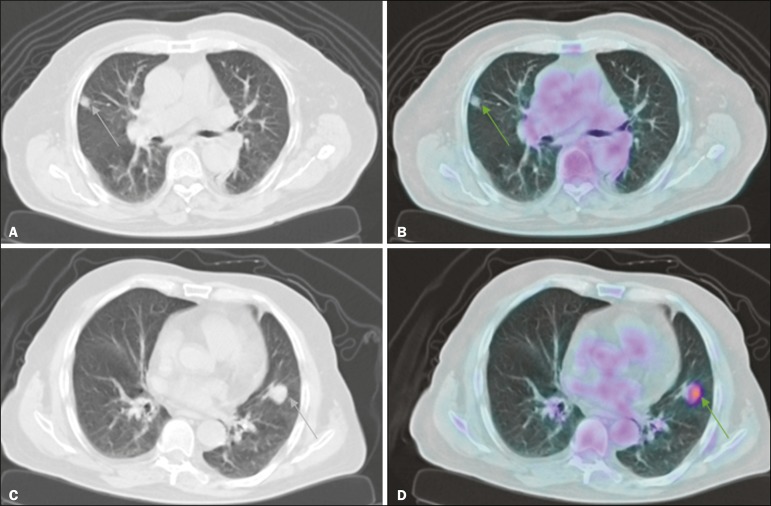
Examples of the use of ^18^F-FDG-PET/CT in SPNs. **A:** A nodule for which the probability of malignancy was 33.2% on the basis of clinical and CT findings, decreasing to 10% when the absence of ^18^F-FDG uptake was taken into account (a follow-up CT scan at two years out showed that the nodule had disappeared). **B:** A nodule for which the probability of malignancy was 6.2% on the basis of clinical and CT findings, increasing to 75.8% when the moderate ^18^F-FDG uptake was taken into account (a subsequent biopsy revealed an adenocarcinoma).

According to the Mayo Clinic model, the probability of malignancy was intermediate in 23 cases (69.7%), low in 2 (0.6%), and high in 8 (2.4%). When the Herder model was applied in those 23 nodules, 6 (26.1%) were reclassified as low and 8 (34.8%) were reclassified as high, whereas 9 (39.1%) were not reclassified (Figure 1). The difference between the Mayo Clinic model and Herder model, in terms of the proportion of cases converted from inconclusive to conclusive, was 36.4% (95% CI: 16.1-56.6%; *p* < 0.01), in favor of the latter model. Of the cases that were classified as having a high or low probability of malignancy by the Mayo Clinic model (Figure 1), none were upgraded and only two were downgraded (from high to intermediate probability) by the Herder model. In addition, when only the cases classified by the Mayo Clinic model as intermediate were considered, that difference increased to 60.9% (95% CI: 40.9-80.8%; *p* < 0.05), and ^18^F-FDG-PET/CT was the determinant of the course of action in 14 cases (60.1%). Figure 2 shows cases that were reclassified on the basis of the ^18^F-FDG-PET/CT findings.

Of the 33 patients included, nine underwent biopsy for histological confirmation, the probability of malignancy having been classified by the Herder model as high in six and as intermediate in three. All of the high-probability cases were malignant, whereas there were benign and malignant findings among the intermediate-probability cases (Table 2). Another five patients underwent a follow-up chest CT examination at our center two years later. In each of the five, the nodule was stable, had shrunk, or had disappeared.

**Table 2:** Detailed description of the 14 SPNs submitted to biopsy or follow-up CT.

Pre-test probability of malignancy according to the Herder model	SPNs for which results were available (n)	Results of a biopsy or follow-up CT
High	6	• 1 small-cell carcinoma • 5 adenocarcinomas of pulmonary origin
Intermediate	5	• 1 benign granuloma • 1 metastasis of a papillary thyroid carcinoma • 1 adenocarcinoma of pulmonary origin • 1 follow-up CT scan, acquired after 2 years, showing considerable nodule shrinkage • 1 follow-up CT scan showing nodule disappearance
Low	3	• 2 follow-up CT scans, acquired after 2 years, showing lesion stability • 1 follow-up CT scan showing disappearance of the nodule

## DISCUSSION

A number of recent studies conducted in Brazil have emphasized the importance of nuclear medicine, especially ^18^F-FDG-PET/CT, for the diagnosis and follow-up of various illnesses^(14-18)^. The present study showed how ^18^F-FDG-PET/CT can aid clinicians during the decision-making process in cases of SPN with an intermediate probability of malignancy. It is noteworthy that ^18^F-FDG-PET/CT modified the probability of malignancy of SPNs in more than half of the cases evaluated, downgrades and upgrades occurring at approximately the same frequency. In addition, the analysis of the cases that underwent biopsy showed that all nodules with a high probability of malignancy had a neoplastic origin. These findings underscore the value of using ^18^F-FDG-PET/CT to define the proper management of cases of SPN.

In cases of SPN, clinical decision-making can be tricky, especially given the high prevalence and varied etiology of the condition^(19)^. In addition, there can be a wide variety of management options, ranging from simple follow-up CT scans to invasive procedures, such as biopsy and surgery^(1,5)^. With that in mind, clinical models were developed to estimate the probability of malignancy in SPNs. One, the Mayo Clinic model, defines the probability of malignancy as low (< 5%), intermediate (between 5% and 65%), or high (> 65%) on the basis of epidemiological and radiological data^(11)^.

Although the management of cases with a high or low probability of malignancy is well-established, the same is not true for cases with an intermediate probability. Biopsy of the SPN can be an alternative in such cases^(1)^. However, histological confirmation of a pulmonary nodule always requires an invasive procedure, with percutaneous access or even thoracotomy, and is subject to complications such as pneumothorax and hemorrhage, with incidence rates of up to 40% and 33%, respectively^(20)^. In addition, the accuracy of an SPN biopsy depends on the location and size of the nodule, and on the technique used, varying from below 50% to almost 100%, depending on the study analyzed^(21)^. Therefore, the use of ^18^F-FDG-PET/CT can help define which patients should undergo biopsy or surgery^(1)^, reducing the number of complications secondary to the procedure.

The use of ^18^F-FDG-PET/CT in SPN can also inform professionals about the best biopsy site when a biopsy is needed^(22)^. Larger nodules may present heterogeneity, with central areas of hypometabolism, and biopsy of the site where there is more intense ^18^F-FDG uptake is recommended. In addition, the quantification of the probability of malignancy is more accurate with ^18^F-FDG-PET/CT^(12)^, as has been demonstrated in other populations^(13,23)^.

In the present study, ^18^F-FDG-PET/CT was able to redefine intermediate probability of malignancy more accurately in 60.9% of the cases (95% CI: 40.9-80.8%; *p* < 0.05). Of those, 26.1% were reclassified as having a low probability, which led to fewer invasive procedures, and 34.8% were reclassified as having a high probability of malignancy, in which case ^18^F-FDG-PET/CT enabled earlier staging in case there was confirmation of malignancy later. Therefore, it is clear that ^18^F-FDG-PET/CT can aid clinical decision-making in the context of an SPN, providing patients with the following benefits: prevention of unnecessary invasive procedures, guiding the biopsy, and early, complete staging.

Among the nine SPNs biopsied, the probability of malignancy was classified as high in six and as intermediate in three. Of the three nodules with an intermediate probability, two had a malignant origin, one being an adenocarcinoma and the other being a metastasis of a papillary thyroid carcinoma-a condition that may present low ^18^F-FDG avidity^(24)^, and the remaining nodule was a benign granuloma. Therefore, when it is not possible to achieve a clear definition of the probability of malignancy for an SPN, it is necessary to use invasive methods to further investigate the nodule, in order to define the most appropriate course of action, despite the decrease in diagnostic accuracy in such cases^(1)^. All cases with a high probability of malignancy had positive biopsies for some type of neoplasm: five were adenocarcinomas and one was a small-cell carcinoma. Among the five cases submitted to radiological follow-up, the Herder model had classified the probability of malignancy as intermediate in three and as low in two; all five patients were doing well. The fact that the results of the biopsies and follow-up PET/CT scans in nodules with an intermediate probability of malignancy showed similar proportions of benign and malignant nodules underscores the need for a more invasive approach or radiological follow-up in such cases.

To use ^18^F-FDG-PET/CT as an auxiliary tool in clinical decision-making, one should be aware of its limitations in the context of SPN. Due to the limited spatial resolution of ^18^F-FDG-PET/CT, its use is not recommended for SPNs smaller than 8 mm^(1,6,25)^. Although the vast majority of pulmonary neoplasms have high ^18^F-FDG avidity, some tumors do not, including mucinous carcinomas^(26)^ or pulmonary metastases, in which the primary tumor also has a low glycolytic activity^(24)^. In addition, inflammatory and infectious pulmonary conditions can have a presentation similar to that of SPN, with high ^18^F-FDG avidity, which results in higher false-positive rates^(27)^. In regions with a high prevalence of granulomatous diseases, this can be a particularly problematic, because it can decrease the specificity of the method^(27,28)^.

It is noteworthy that, in the present study none of the SPNs classified as having a high probability of malignancy were found to be caused by infectious or inflammatory processes. Most of the patients who undergo ^18^F-FDG-PET/CT scans in Brazil have private health insurance plans^(29)^ because they belong to the higher socioeconomic classes that are therefore at a lower risk of developing tuberculosis^(30)^ and other infectious respiratory diseases^(31)^. That was also observed in another study, conducted at a private hospital in Brazil, in which nearly 93% of the SPNs with an SUVmax > 2.5 were malignant^(32)^. Therefore, to optimize the use of ^18^F-FDG-PET/CT in SPNs, it is important to consider other patient epidemiological data regarding the presence of infectious lung diseases.

Other limitations of ^18^F-FDG-PET/CT in the investigation of SPN are the limited availability and high cost of the method. In Brazil, access to ^18^F-FDG-PET/CT for the investigation of SPN is limited and is quite heterogeneous among the different regions of the country^(29)^.

## CONCLUSION

This study showed that the use of ^18^F-FDG-PET/CT changed the pre-test probability of malignancy classification of more than half of the SPNs classified as intermediate by a model that used patient clinical and radiological data only, confirming that ^18^F-FDG-PET/CT is an important tool to aid clinical decision-making in such cases.
